# A Recombinant Newcastle Disease Virus (NDV) Expressing S Protein of Infectious Bronchitis Virus (IBV) Protects Chickens against IBV and NDV

**DOI:** 10.1038/s41598-018-30356-2

**Published:** 2018-08-10

**Authors:** Edris Shirvani, Anandan Paldurai, Vinoth K. Manoharan, Berin P. Varghese, Siba K. Samal

**Affiliations:** Virginia-Maryland College of Veterinary Medicine, University of Maryland, College Park, MD USA

**Keywords:** Genetic engineering, Immunoblotting, Live attenuated vaccines

## Abstract

Infectious bronchitis virus (IBV) causes a highly contagious respiratory, reproductive and urogenital tract disease in chickens worldwide, resulting in substantial economic losses for the poultry industry. Currently, live-attenuated IBV vaccines are used to control the disease. However, safety, attenuation and immunization outcomes of current vaccines are not guaranteed. Several studies indicate that attenuated IBV vaccine strains contribute to the emergence of variant viruses in the field due to mutations and recombination. Therefore, there is a need to develop a stable and safe IBV vaccine that will not create variant viruses. In this study, we generated recombinant Newcastle disease viruses (rNDVs) expressing the S1, S2 and S proteins of IBV using reverse genetics technology. Our results showed that the rNDV expressing the S protein of IBV provided better protection than the rNDV expressing S1 or S2 protein of IBV, indicating that the S protein is the best protective antigen of IBV. Immunization of 4-week-old SPF chickens with the rNDV expressing S protein elicited IBV-specific neutralizing antibodies and provided complete protection against virulent IBV and virulent NDV challenges. These results suggest that the rNDV expressing the S protein of IBV is a safe and effective bivalent vaccine candidate for both IBV and NDV.

## Introduction

Infectious Bronchitis (IB) is an acute and highly contagious viral respiratory disease of chickens^1,2^. IB causes major economic losses in commercial chickens throughout the world^1,3^. It is one of the most prevalent diseases in the poultry industry. The disease is usually characterized by respiratory signs including ocular and nasal discharges, gasping, coughing and sneezing. However, the virus can also infect urogenital and reproductive tracts causing renal dysfunction and decreased egg production^3,4^.

IB is caused by infectious bronchitis virus (IBV), a member of the family *Coronaviridae*. The genome of IBV is a single stranded, positive-sense RNA of approximately 27.6 kb in length. It encodes five major structural proteins: spike (S), small envelope (E), membrane (M), nucleocapsid (N) and fifteen non-structural proteins. The S glycoprotein is the major antigen against which neutralizing and protective antibodies are produced. The S protein is partially or completely cleaved into the amino-terminal S1 and to the carboxy-terminal S2 subunits post translationally by a furine-like protease of host^4,5^. The S1 subunit is highly variable among different isolates of IBV. It is responsible for viral attachment to host cell and contains major neutralizing epitopes. The S2 subunit is highly conserved among IBV strains and contributes to viral fusion activity and elicits some minor but broadly reactive neutralizing antibodies^5–10^.

Currently IB in commercial chickens is controlled by the use of live-attenuated and inactivated IBV vaccines. However, safety, attenuation and immunization outcomes of current live-attenuated vaccines which, were developed by serial passaging of field isolates of IBV in chicken embryonated eggs, are not guaranteed, particularly with regards to the age at immunization, vaccine dose, type of bird and route of vaccination^11^. Live vaccines have been found as one of the sources of IB outbreaks^12^. Live-attenuated vaccine strains also are not genetically stable and have a tendency to revert back to virulence^13,14^. Furthermore, circulation of live-attenuated viruses in the environment provides a setting in which the viral population may undergo mutations and recombination leading to creation of variant viruses^15,16^. Moreover, live IBV vaccines may cause pathological lesions or secondary bacterial infections in young vaccinated chicks^17,18^. Inactivated IBV vaccines, which usually are administered by injection to layers and breeders are not an alternative for live-attenuated IBV vaccines. Inactivated vaccines of IBV do not elicit strong immune responses in chickens against circulating virulent IBV strains^19,20^. The production and administration of inactivated vaccines are also time consuming and costly^13,14^. Therefore, there is a great need to develop an alternative IBV vaccine.

Development of viral vectored vaccines to control IB is an alternative approach. One of the important advantages of vectored IBV vaccines is that it will not create variant viruses, which is a major drawback of the current live-attenuated IBV vaccines. Several viruses, such as, fowl pox virus, adenovirus and Newcastle disease virus (NDV) have been evaluated as vaccine vectors for IBV^21–25^. Among these viral vectors NDV holds great promise, because it induces effective local and systemic immune responses, it is a safe vaccine vector, and it will serve as a bivalent vaccine against IBV and NDV.

NDV belongs to the genus *Avulavirus* in the family *Paramyxoviridae*. Virulent NDV strains cause a fatal neurological disease in chickens. NDV strain LaSota has been used as a safe and effective live vaccine for more than 60 years^26,27^. Recombinant NDV strain LaSota has been evaluated as a vaccine vector against several avian pathogens including IBV^28–32^. In one study, rNDV expressing S2 protein of IBV was found to induce partial protection against a virulent IBV challenge^23^. In another study, it was shown that rNDV expressing S1 protein of IBV resulted in inducing protective immunity against virulent IBV challenge^24^. However, the protective efficacy of the whole S protein of IBV expressed by rNDV vector has not been evaluated and it is also not known among S1, S2 and S proteins of IBV, which is the best protective antigen for the development of viral vectored IBV vaccines. In this study, we have used reverse genetics to generate rNDVs expressing S1, S2 and S proteins of IBV strain Mass-41. The protective efficacies of rNDVs expressing S1, S2 or S protein of IBV were compared in chickens against a virulent IBV strain Mass-41 challenge. Our results showed that the whole S protein is the best protective antigen of IBV. We also showed that rNDV strain LaSota expressing the S protein of IBV strain Mass-41 protects chickens against both virulent IBV and virulent NDV challenges.

## Result

### Generation of rNDVs expressing S1, S2 or S protein of IBV

**Figure 1:**
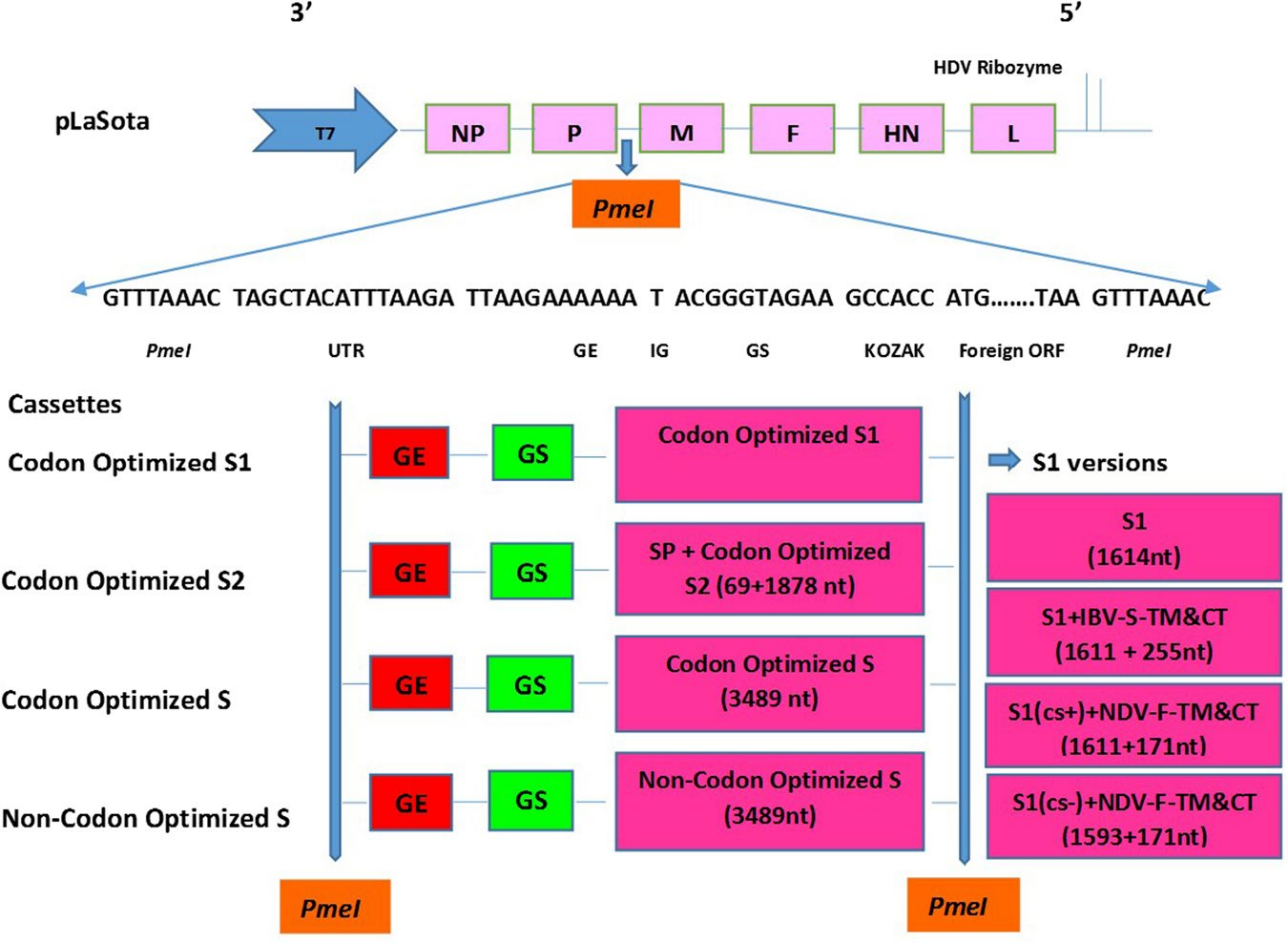
Schematic diagram of recombinant NDV constructs containing IBV genes. Seven transcription cassettes including; 1–4) Four versions of codon optimized S1 subunit of S gene of IBV strain Mass-41; namely, **(a)** S1 subunit of S gene (1614 nt), **(b)** S1 subunit of S gene (1611 nt) fused with N-terminus of transmembrane and cytoplasmic tail of S gene (255 nt), **(c)** S1 subunit of S gene (1611 nt) containing five putative cleavage site residues of S gene fused with N-terminus of transmembrane and cytoplasmic tail of F gene of NDV (171 nt). In this construct, five C-terminus putative cleavage site residues of S1 gene (RRFRR) plus the first serine (S) residue of N-terminus of transmembrane and cytoplasmic tail of F gene of NDV provides six putative cleavage site residues of S protein of IBV strain Mass-41 (RRFRR/S). **(d)** S1 gene (1593 nt) without cleavage site residues of S gene fused with N-terminus of transmembrane and cytoplasmic tail of F gene of NDV (171 nt), 5) the N-terminus of codon optimized S2 gene of IBV strain Mass-41 (1878 nt) fused with C-terminus of signal peptide sequence of S gene (69 nt), 6) the codon-optimized S gene (3489 nt) and 7) the non-codon optimized S gene of IBV strain Mass-41 (3489 nt) were flanked into individual plasmids containing cDNA of LaSota between P and M genes using *PmeI* site. Each transcription cassette contains the ORF of foreign gene with the addition of *PmeI* restriction enzyme site sequence, 15 nt of NDV UTR, GE signal of NDV, one T nucleotide as intergenic sequence, GS signal of NDV, nucleotides for maintaining the rule of six and Kozak sequence.

The expression cassettes containing the codon optimized S1, S2, S and non-codon optimized S genes of IBV were cloned into the cDNA encoding the complete antigenome of NDV strain LaSota, using the *PmeI* site, between P and M genes (Fig. 1). The correct sequences of genes cloned into full length cDNA of NDV were confirmed by nucleotide sequence analysis. Infectious recombinant NDVs containing S1, S2 and S genes of IBV were recovered from all cDNAs. The sequences of S1, S2 and S genes present in the rNDVs were confirmed by RT-PCR. To evaluate genetic stability of rNDV expressing codon optimized S protein, the viruses were passaged five times in 9-day-old embryonated specific pathogen free (SPF) chicken eggs. The nucleotide sequence analysis of the S gene showed that the inserted ORF were maintained without any adventitious mutations.

### Evaluation of the expression of the S1, S2 and S proteins of IBV

**Figure 2:**
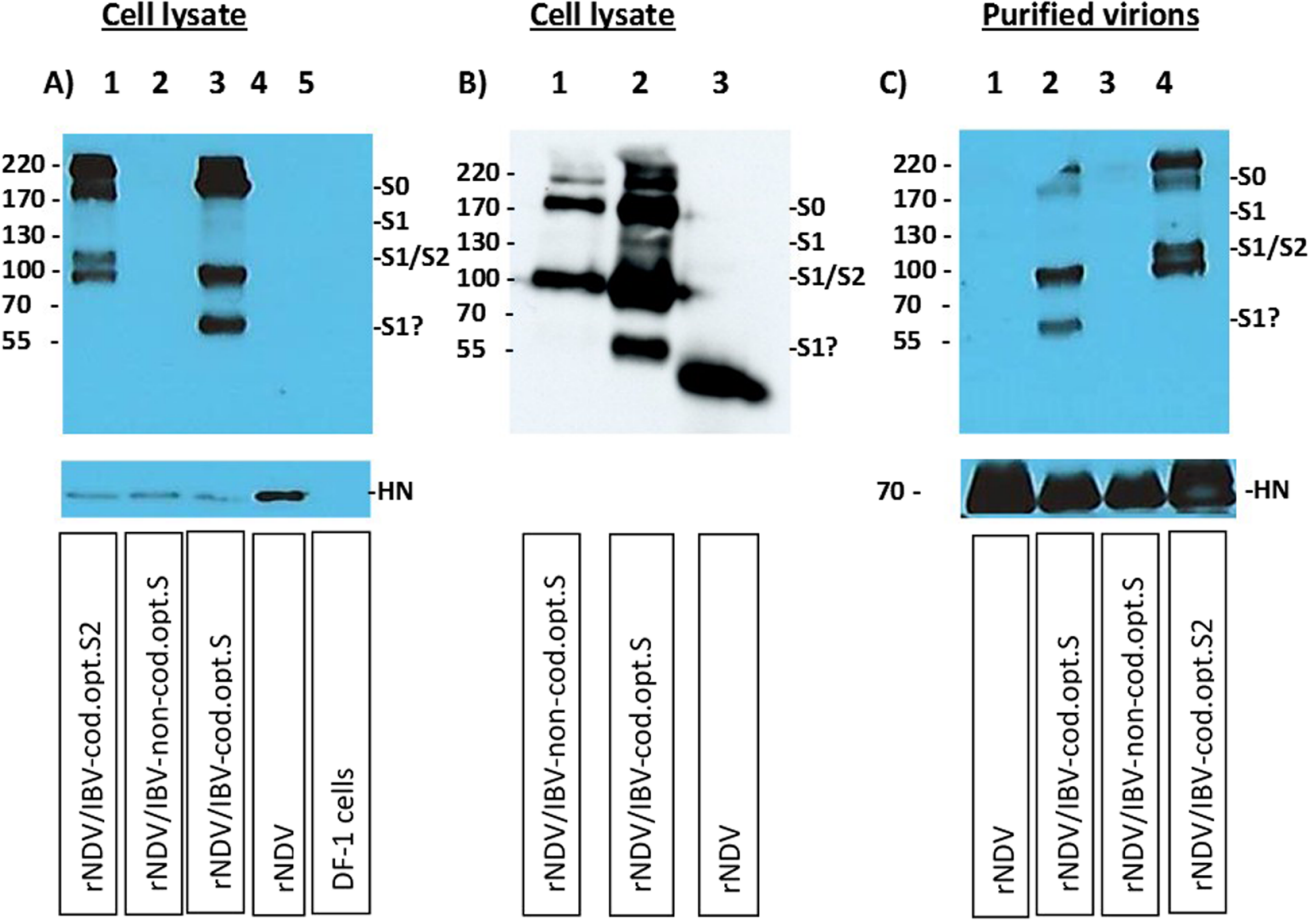
Western blot analysis of rNDVs expressing S or S2 protein of IBV. The expression of codon optimized S, and S2 proteins and non-codon optimized S protein of IBV were detected by Western blot analysis in infected DF-1 cell lysates, using a chicken polyclonal anti IBV serum (A-upper panel & B). The panel B was added to show the expression of non-codon optimized S protein clearly, since the expression of non-codon optimized S protein was not clear in panel A. For the codon optimized S protein of IBV expressed from rNDV (A-lane 3 and B-lane 2) two bands on top (~170–220 kDa) represent uncleaved S protein (S0) or polymeric forms of S1 or S2 protein. The ~130 kDa band, ~95 kDa band and ~60 kDa band represent S2 or S1 subunit of cleaved S protein. In the case of non-codon optimized S protein expressed from rNDV (2A-lane 2 and B-lane 1), two bands on top (~170–220 kDa) represent uncleaved S protein (S0) or polymeric forms of S2 or S1 protein and ~95 kDa band represents S2 or S1 subunit of cleaved S protein. In the case of rNDV/IBV- S2 (A-lane 1), there are two bands (~170–220 kDa) on top, representing polymeric forms of S2 protein, the ~105 kDa and the ~95 kDa band representing S2 subunit. Lane 4 of panel A and lane 3 of panel B represent rNDV as control. Lane 5 of panel A represents non-infected DF-1 cells. The incorporation of codon optimized S2 and S proteins and non-codon optimized S protein of IBV in NDV particles were detected by Western blot (C-upper panel). Two bands (~170–220 kDa) on top represent uncleaved S protein (S0) or polymeric forms of S2 or S1 protein (C-lane 2). The ~95 kDa and the ~60 kDa band represent S2 or S1 subunit of cleaved S protein (C-lane 2). The two bands (~170–220 kDa) on top represent polymeric forms of S2 protein, the ~105 kDa band and the ~95 kDa band represent S2 subunit(C-lane 4). The lane 1 and 3 of panel C represent purified rNDV control and purified rNDV expressing non-codon optimized S protein, respectively. A monoclonal anti-NDV/HN antibody was used to detect the 70 kDa of HN protein of NDV in DF-1 cell lysates (A-lower panel); rNDV/S2 (lane 1), rNDV/IBV-non-cod.opt.S (lane 2), rNDV/IBV- cod.opt.S (lane 3), rNDV (lane 4) and incorporated in NDV particles (C-lower panel); rNDV (lane 1), rNDV/IBV-cod.opt.S (lane 2), rNDV/IBV-non-cod.opt.S (lane 3) and rNDV/S2 (lane 4). The full-length gels are presented in supplementary Figure S1.

The expression of codon optimized S2, and S proteins and non-codon optimized S protein of IBV strain Mass-41 by rNDV constructs was detected by Western blot analysis in DF-1 cells using a chicken polyclonal anti IBV serum (Fig. 2A-upper panel and B). As the expression of non-codon optimized S was not detected clearly in the first attempt (Fig. 2A), we detected it in another attempt (Fig. 2B). The expression level of codon optimized S protein of IBV was significantly higher than that of the non-codon optimized S protein of IBV. For the codon optimized S protein of IBV expressed from rNDV (Fig. 2A-Lane 3 and Fig. 2B - lane 2), the two bands on top (~170–220 kDa) probably represent either uncleaved S protein (S0) or polymeric forms of S protein. The ~130 kDa, the ~95 kDa and the ~60 kDa band represent S1 or S2 subunit of cleaved S protein of IBV. In the case of non-codon optimized S protein of IBV expressed from rNDV (Fig. 2A-lane 2 and Fig. 2B-lane 1), the two bands on top (~170–220 kDa) probably represent uncleaved S protein (S0) or polymeric forms of S protein. The ~95 kD band represents S2 or S1 subunit of cleaved S protein of IBV. In the case of rNDV/IBV- S2 strain (Fig. 2A-lane 1), there are two bands (~170–220 kDa) on top, representing polymeric folded forms of S2 protein, a ~105 kDa band and a ~95 kDa band representing S2 subunit. The expression of S2 protein from a transcription cassette in which the signal peptide sequences of S protein was not fused with S2 gene was not detected (data not shown). Lane 4 of Fig. 2A and lane 3 of Fig. 2B represent rNDV as control. Lane 5 of panel A represents non-infected DF-1 cells. These results showed that codon optimized S and S2 proteins of IBV were expressed efficiently. The non-codon optimized S protein was also expressed from rNDV, but not efficiently and not consistently. A monoclonal anti-NDV/HN antibody was used to detect a ~70 kDa of HN protein of NDV in lysates, confirming similar level of NDV protein in each lane (Fig. 2A-lower panel). We further evaluated incorporation of IBV S and S2 proteins into NDV virions. The rNDVs expressing codon optimized S and S2 proteins and rNDV expressing non-codon optimized S protein were inoculated into eggs, 3 days after inoculation, viral particles in infected allantoic fluid were partially purified and analyzed by Western blot (Fig. 2C-upper panel). Two bands (~170–220 kDa) on top, representing S protein, a ~95 kDa band and a ~60 kDa band representing S2 or S1 subunit of cleaved S protein, were detected in purified particles of rNDV expressing codon optimized S protein by Western blot analysis (Fig. 2C-lane 2). The lane 4 of Fig. 2C shows two bands (~170–220 kDa) on top, representing polymeric folded of S2 protein, a ~105 kDa band and a ~95 kDa band representing S2 subunit. The lane 1 of Fig. 2C represents purified rNDV control and lane 3 of Fig. 2C shows purified rNDV expressing non-codon optimized S protein. These results suggested that the codon optimized S and S2 proteins of IBV expressed by rNDVs were incorporated into rNDV particles. A monoclonal anti-NDV/HN antibody was used to detect a ~70 kDa of HN protein of NDV in partially purified virions, confirming similar level of NDV protein in each lane (Fig. 2C-lower panel).

**Figure 3:**
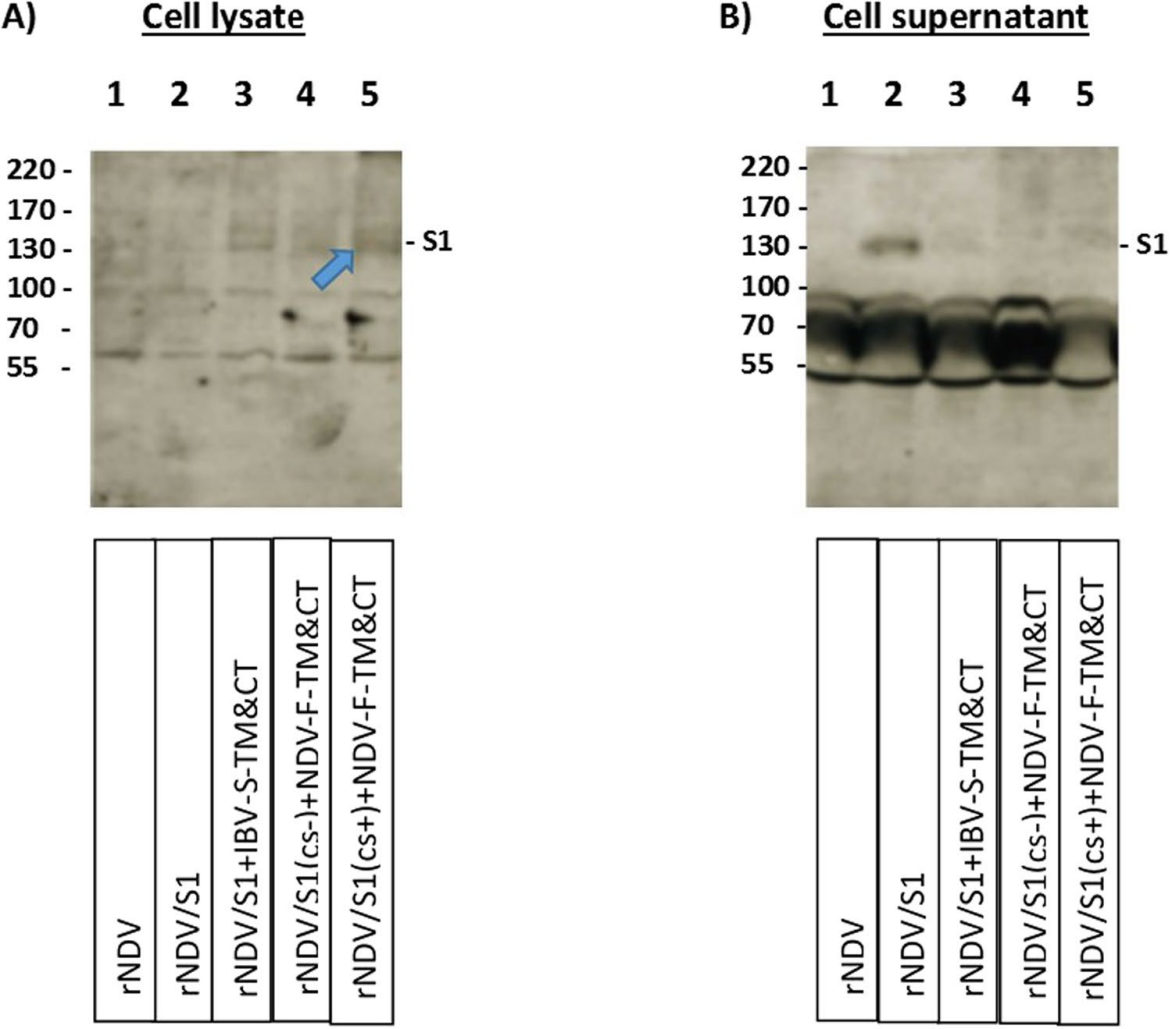
Western blot analysis of rNDV expressing S1 protein of IBV. The expression of codon optimized S1 protein of IBV expressed from four individual rNDVs expressing four different expression cassettes of S1 protein were detected using Western blot in cell lysates **(A)** and cell supernatant **(B)** of infected DF-1 cells infected with rNDVs, using a chicken polyclonal anti IBV serum. The lanes 1–5 represent cell lysates of rNDV, rNDV/S1, rNDV/S1 + IBV-S-TM&CT, rNDV/S1(cs−)+NDV-F-TM&CT, rNDV/S1(cs+) + NDV-F-TM&CT, respectively. A ~130 kD band represent expression of S1 protein by rNDV/S1 + IBV-S-TM&CT, rNDV/S1(cs−) + NDV-F-TM&CT and rNDV/S1(cs+) + NDV-F-TM&CT in infected DF-1 cell lysate (A lanes 3–5) and rNDV/S1 in infected DF-1 cell supernatant (B-lane 2). The full-length gel is presented in Supplementary Figure S1.

The expression of codon optimized S1 protein expressed from four individual rNDV constructs were detected by Western blot analysis in lysates (Fig. 3A) and supernatant (Fig. 3B) of infected DF-1 cells, using a chicken polyclonal anti IBV serum. The lanes 1–5 represent infected DF-1 cell lysates of rNDV, rNDV/S1, rNDV/S1 + IBV-S-TM&CT, rNDV/S1(cs−) + NDV-F-TM&CT and rNDV/S1(cs+) + NDV-F-TM&CT, respectively. A ~130 kDa band representing expression of S1 by rNDV/S1 + IBV-S-TM&CT, rNDV/S1(cs−) + NDV-F-TM&CT, and rNDV/S1(cs+) + NDV-F-TM&CT in lysate of DF-1 cells (Fig. 3A-lanes 3–5) and rNDV/S1 in infected DF-1 cell supernatant (Fig. 3B-lane 2) was observed. Our attempts to detect the incorporation of the S1 protein into NDV envelope were not successful, due to the difficulties in the detection of very low level of S1 protein by Western blot analysis (data not shown). Our results showed that the S1 protein was expressed at very low level by all the rNDVs based on Western blot analysis. Only the unmodified S1 protein was detected in the cell culture supernatant.

### Growth characteristics of rNDV constructs

**Figure 4:**
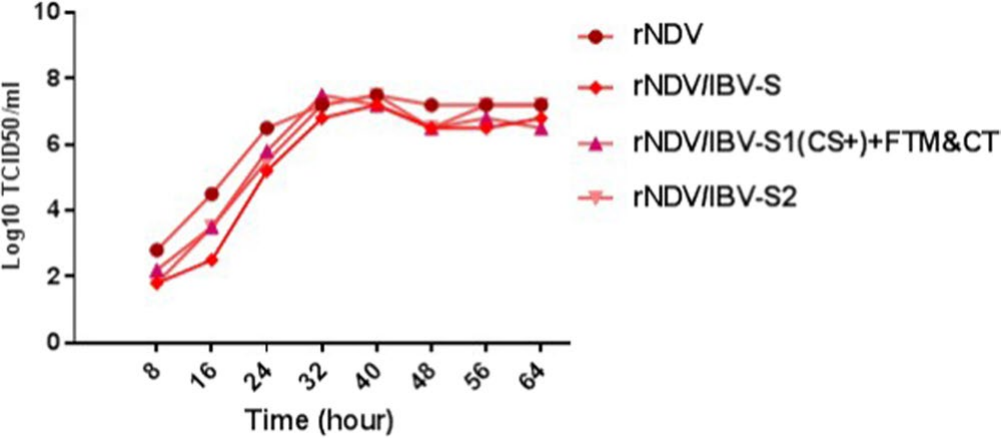
Multicycle growth kinetics of the rNDV constructs in DF-1 cells. DF-1 cells were infected with each recombinant virus at a MOI of 0.1. Two hundred µl of supernatant from infected cells were collected and replaced with fresh DMEM including 10% normal allantoic fluid at 8 hours intervals. The titer of the virus in harvested samples were determined by TCID_50_ assay in DF-1cell.

The recovered rNDVs were passaged in 9-day-old embryonated SPF chicken eggs. All the viruses were able to replicate well in eggs (≥2^8^ HAU/ml). rNDV/S1, rNDV/S1(cs+) + NDV-F-TM&CT, rNDV/S2, rNDV/codon optimized-S and rNDV were evaluated in the presence of exogenous protease in DF-1 cells (Fig. 4). Compared to the parental virus, rNDV expressing codon optimized S protein of IBV grew slightly less efficiently. The maximum titer of parental virus reached 10^7.5^ TCID_50_/ml at 40 hours post infection, whereas the maximum titer of rNDV expressing codon optimized S gene of IBV reached 10^7.2^ TCID_50_/ml at 40 hours post infection. These results indicated that presence of S, S1 and S2 genes did not significantly affect the growth characteristics of rNDV.

### The protective efficacy of rNDVs expressing S1, S2 or S protein of IBV in chickens against a virulent IBV challenge

#### IBV protection experiment 1

**Figure 5:**
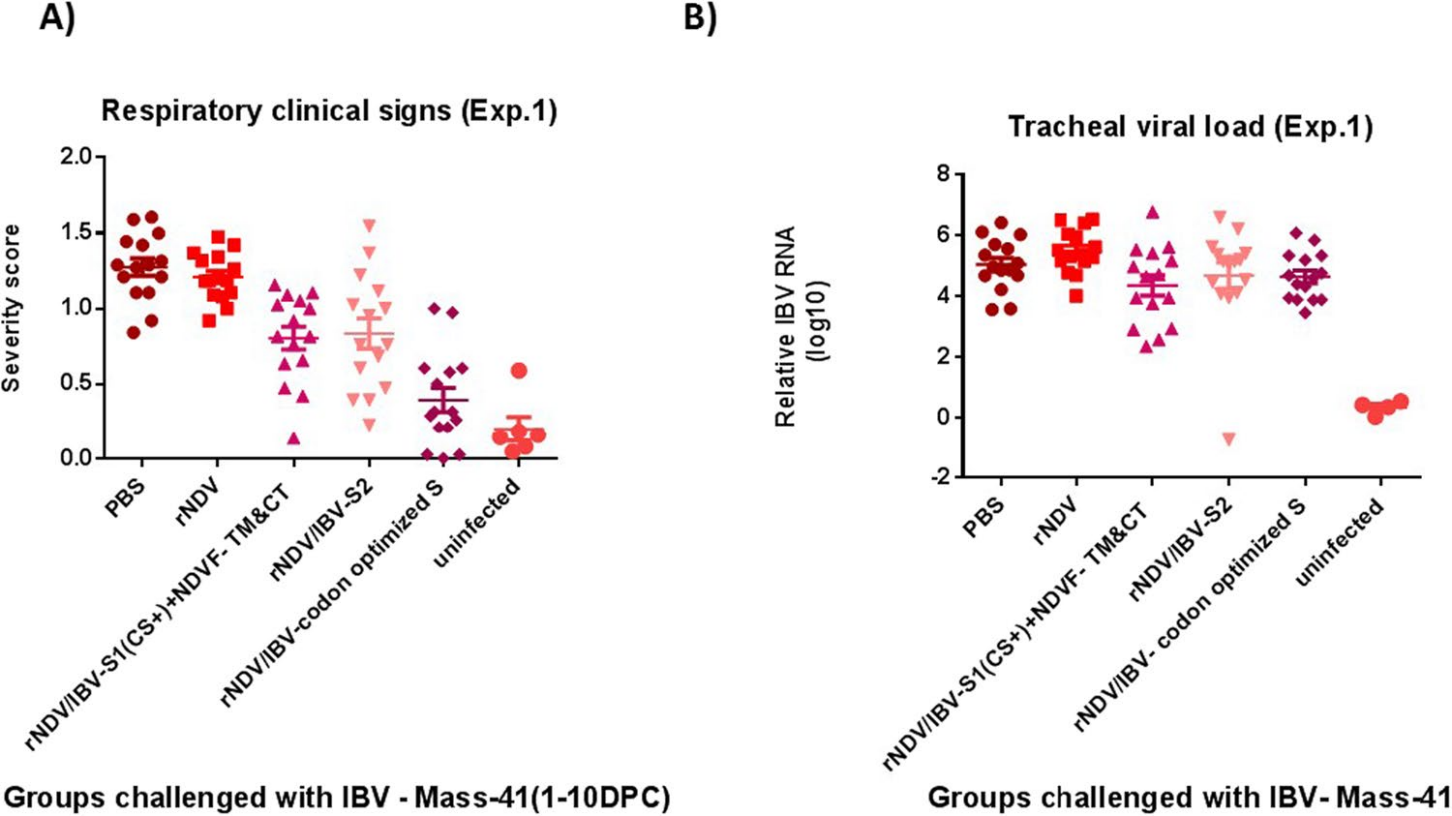
The protective efficacy of rNDV constructs in one-day-old immunized SPF chickens against virulent IBV challenge (IBV protection experiment 1) at 21 days post-immunization. **(A)** Respiratory clinical signs of IBV following challenge with a 10^3.1^ EID_50_ of virulent IBV strain Mass-41. The immunized chickens were challenged with a virulent IBV strain Mass-41. The severity scores of IBV clinical signs include; ocular discharge, nasal discharge and difficulty in breathing (0 = normal, 1 = presence of mild ocular discharge, mild nasal discharge and or sneezing 2 = presence of heavy ocular discharge and or heavy nasal discharge with mild tracheal rales and mouth breathing and or coughing 3 = heavy ocular discharge and heavy nasal discharge with sever tracheal rales and mouth breathing, gasping, dyspnea and or severe respiratory distress) were recorded twice a day for each chicken for 10 days after challenge. The severity scores represent as average scores of clinical signs measured for each chicken over 10 days. **(B)** Relative viral load determined by RT-qPCR in tracheal swab samples at day five following virulent IBV challenge. The relative viral load expressed as mean reciprocal ± SEM log 10.

To evaluate the protective efficacy of rNDVs expressing S1, S2 or S protein of IBV, SPF chicks were immunized at 1-day-old age with each virus via oculanasal (ON) route. At three weeks post-immunization, chickens were challenged with virulent IBV strain Mass-41. The severity scores of IBV clinical signs were recorded twice a day for 10 days post-challenge (Fig. 5A). Compared to chickens immunized with parental rNDV and chickens inoculated with PBS, chickens immunized with rNDVs expressing codon optimized S, S1 or S2 protein of IBV showed significantly less severe of clinical signs (P < 0.05). Among groups of chickens immunized with rNDVs expressing codon optimized S1, S2 or S protein, the group immunized with rNDV expressing codon optimized S protein showed the least severity of clinical signs (P < 0.05). In order to evaluate the efficacy of rNDVs expressing S1, S2 or S protein of IBV in preventing shedding of virulent IBV challenge virus in immunized chickens, on day five post-challenge, tracheal swab samples were collected from chickens of each group and were evaluated for the viral load by RT-qPCR. Our results did not show significant difference in virus shedding among groups of immunized chickens at day five post challenge (Fig. 5B). However, the results of the inoculation of the tracheal swab samples into 10-day-old embrynated chicken eggs showed that 14 out of 15 (93.3%) chickens vaccinated with rNDV expressing codon optimized S protein of IBV and 0 out of 5 (0%) of non-infected chickens were shedding virus in trachea, respectively, whereas 15 out of 15 (100%) of chickens of all other groups were shedding virus in the trachea (data not shown).

#### IBV protection experiment 2

**Figure 6:**
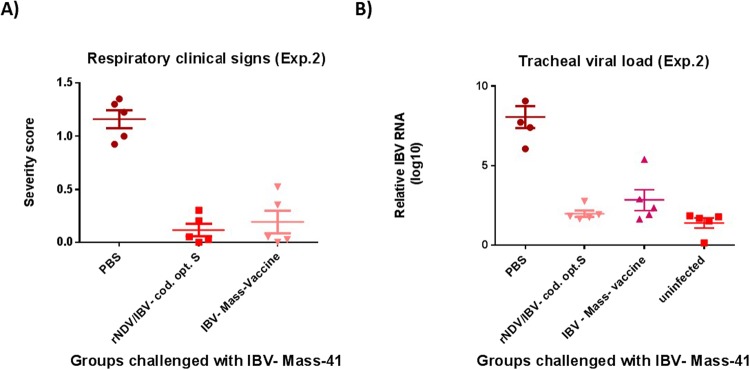
The protective efficacy of rNDV expressing codon optimized S protein of IBV against IBV challenge in immunized SPF chickens at 4-week-old age. **(A)** Respiratory clinical signs of IBV following challenge with virulent IBV strain Mass-41. Three weeks after immunization, chickens were challenged with 10^3.1^ EID_50_ of a virulent IBV strain Mass-41. The severity scores of IBV clinical signs, described in the legend of Fig. 1, were recorded twice a day for each chicken for 10 days after challenge. The severity score represents as average score of clinical signs measured for each chicken over 10 days. **(B)** Relative viral load determined by RT-qPCR in tracheal swab samples at day 5 following virulent IBV challenge. The relative viral load expressed as mean reciprocal ± SEM log 10.

To evaluate the protective efficacy of rNDV expressing codon optimized S protein of IBV in adult chickens, SPF chickens were immunized at 4-week-old age. The protective efficacy of rNDV expressing codon optimized S gene of IBV was determined by challenging the immunized chickens with the World Organization for Animal Health (OIE) recommended dose (10^3.1^ EID_50_) of virulent IBV strain Mass-41 at 3 week post-immunization^1^. The severity scores of IBV clinical signs were recorded twice a day for 10 days post-challenge (Fig. 6A). Compared to chickens inoculated with PBS, chickens immunized with rNDV expressing codon optimized S protein of IBV and chickens immunized with a commercial live attenuated IBV vaccine showed significantly less severe clinical signs (P < 0.05). In order to evaluate the efficacy of rNDV expressing S protein of IBV in preventing shedding of virulent IBV in immunized chickens, at day 5 following challenge with a virulent IBV, the tracheal swab samples collected from five chickens of each group were analyzed for the viral load by RT-qPCR. Our results showed that chickens vaccinated with rNDV expressing codon optimized S protein of IBV and chickens vaccinated with a commercial IBV vaccine showed very low levels of viral load in the trachea, whereas chickens inoculated with PBS showed high levels of viral load in the trachea (P < 0.05). However, compared to chickens immunized with a commercial IBV vaccine, chickens immunized with rNDV expressing codon optimized S showed slightly less viral load in the trachea (Fig. 6B).

#### IBV protection experiment 3

**Figure 7:**
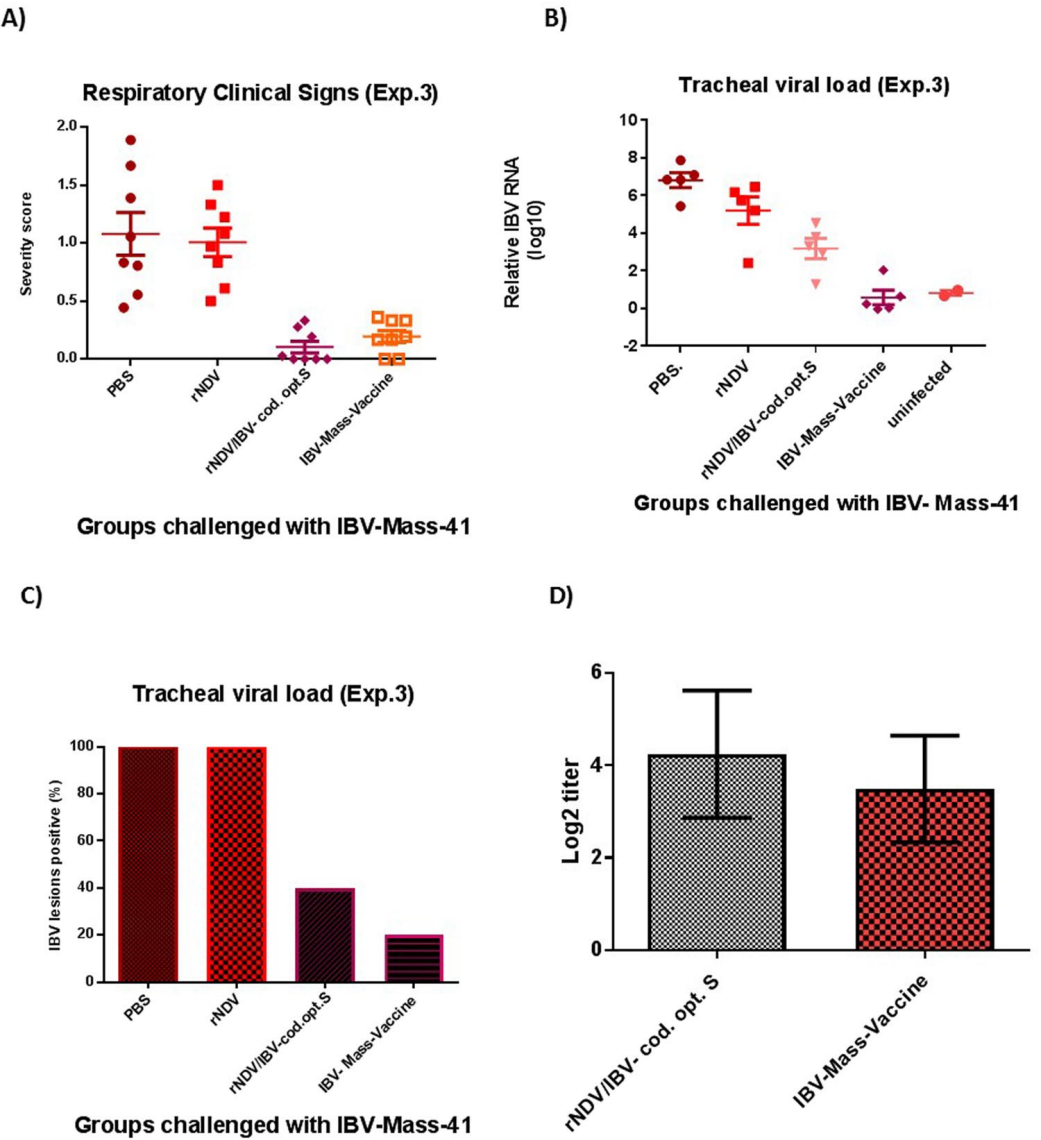
The protective efficacy of rNDV expressing codon optimized S protein of IBV and neutralizing antibody response against high dose of IBV challenge in immunized SPF chickens at 4-week-old age. **(A)** Respiratory clinical signs of IBV following challenge with virulent IBV strain Mass-41. Three weeks after immunization, chickens were challenged with 10^4.7^ EID_50_ of a virulent IBV strain Mass-41. The severity scores of IBV clinical signs, described in the legend of Fig. 1, were recorded twice a day for each chicken for 8 days after challenge. The severity score represents as average score of clinical signs measured for each chicken over the 8 days. **(C)** Relative viral load determined by RT-qPCR in tracheal swab samples at day 4 following virulent IBV challenge. The relative viral load expressed as mean reciprocal ± SEM log 10. **(D)** Each tracheal fluid was tested for IBV specific lesions on chicken embryo by inoculation (0.1 ml) of one 10-day-old embryonated SPF chicken egg. **(D)** Neutralizing antibody response against IBV. Antibodies induced against IBV were assessed using virus neutralization assay. Serum titers are expressed as reciprocals Log 2 dilution.

To evaluate the protective efficacy of rNDV expressing codon optimized S protein of IBV in adult chickens against a higher dose of virulent IBV challenge, SPF chickens were immunized at 4-week-old age. The protective efficacy of rNDV expressing codon optimized S gene of IBV was determined by challenging the immunized chickens with 10^4.7^ EID_50_ virulent IBV strain Mass-41 at 3 week post-immunization. The severity scores of IBV clinical signs were recorded twice a day for 8 days post-challenge (Fig. 7A). Compared to chickens immunized with rNDV and chickens inoculated with PBS, chickens immunized with rNDV expressing codon optimized S protein of IBV and chickens immunized with a commercial live attenuated IBV vaccine showed significantly less severe clinical signs (P < 0.05). In order to evaluate the efficacy of rNDV expressing S protein of IBV in preventing shedding of virulent IBV in immunized chickens, at days 4 following challenge with virulent IBV, the tracheal swab samples collected from five chickens of each group were analyzed for the IBV specific lesions in chicken embryo. Our results showed that 2 out of 5 (40%) chickens vaccinated with rNDV expressing codon optimized S protein of IBV and 1 out of 5 (20%) chickens vaccinated with a commercial IBV vaccine were shedding virus in trachea, respectively, whereas 5 out of 5 (100%) of chickens immunized with parental rNDV and 5 out of 5 (100%) of chickens inoculated with PBS were shedding virus in the trachea (Fig. 7C). The tracheal swab samples collected from five chickens of each group were also analyzed for the viral load by RT-qPCR. Our results showed that chickens vaccinated with rNDV expressing codon optimized S protein of IBV showed low levels of viral load in the trachea and chickens vaccinated with a commercial IBV vaccine showed very low levels of viral load in the trachea, whereas chickens inoculated with PBS and rNDV showed high levels of viral load in the trachea. Compared to chickens immunized with rNDV expressing codon optimized S protein, chickens immunized with a commercial IBV vaccine showed less viral load in the trachea (P < 0.05) (Fig. 7B).

#### IBV protection experiment 4

**Figure 8:**
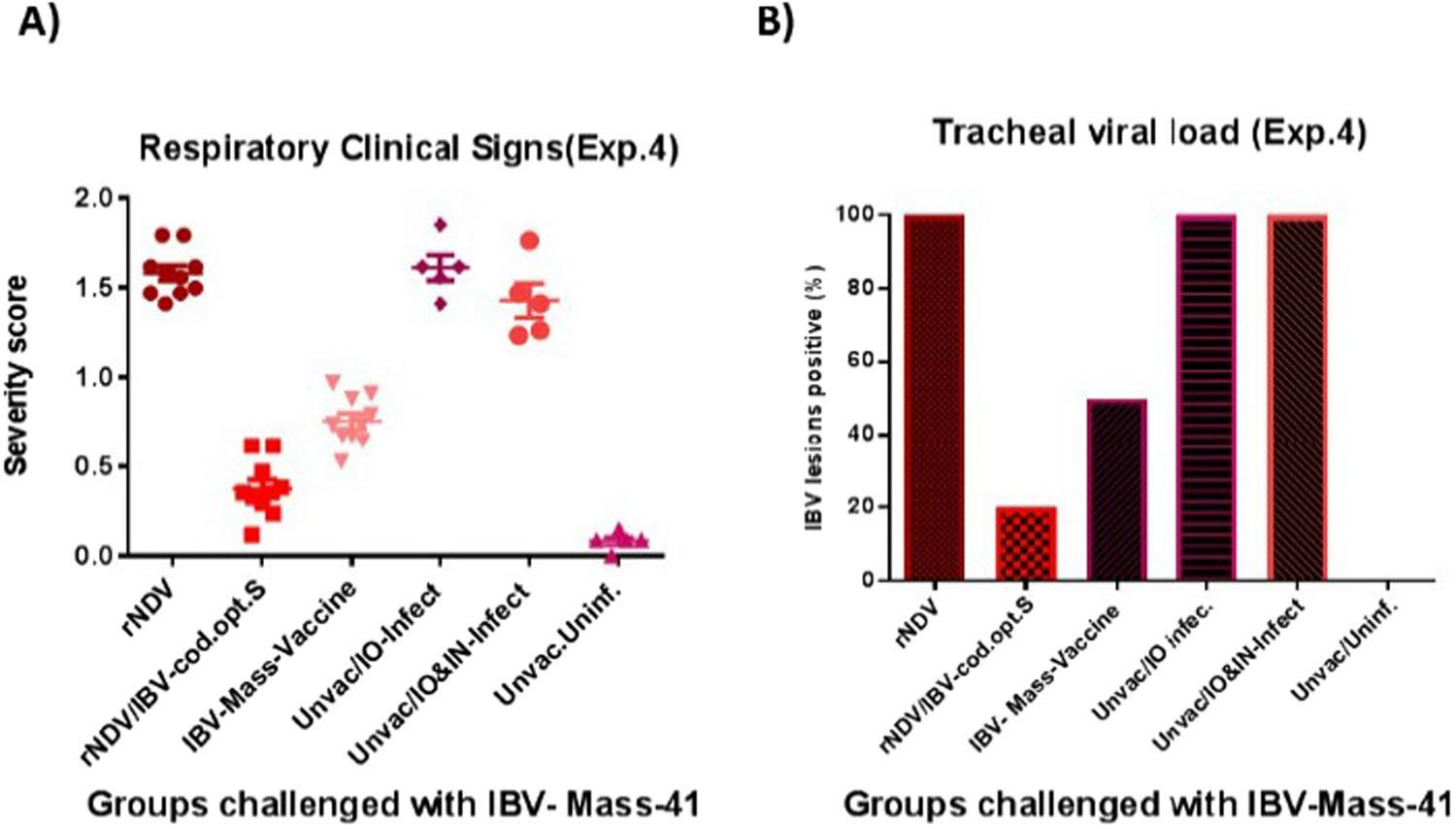
The protective efficacy of rNDV expressing codon optimized S protein of IBV against IBV challenge in immunized SPF chicks at 1-day-old age, by the intraocular (IO) challenge route. **(A)** Respiratory clinical signs of IBV following challenge with virulent IBV strain Mass-41. Three weeks after immunization, chickens were challenged with 10^4^ EID_50_ of a virulent IBV strain Mass-41 by the intraocular route. The severity scores of IBV clinical signs, described in the legend of Fig. 1, were recorded twice a day for each chicken for 10 days after challenge. The severity score represents as average score of clinical signs measured for each chicken over 10 days. **(B)** Each tracheal fluid was tested for IBV specific lesions on chicken embryo by inoculation (0.2 ml) of five 10-day-old embryonated SPF chicken egg. IN = intranasal

To evaluate the effect of the route of inoculation of virulent IB challenge virus on the outcomes of the protective efficacy of rNDV expressing codon optimized S protein of IBV, SPF chicks were immunized at 1-day-old age. The protective efficacy of rNDV expressing codon optimized S gene of IBV was determined by challenging the immunized chickens with 10^4^ EID_50_ virulent IBV strain Mass-41 by the intraocular route at 3 week post-immunization. This route of challenge has been specified in USDA-CFR-9 for IBV^33^. The severity scores of IBV clinical signs were recorded twice a day for 10 days post-challenge. Compared to chickens immunized with rNDV and unvaccinated chickens, chickens immunized with rNDV expressing codon optimized S protein of IBV and chickens immunized with a commercial live attenuated IBV vaccine showed significantly less severe clinical signs. However, compared to chickens immunized with commercial IBV vaccine, chickens immunized with rNDV expressing S protein showed less severe clinical signs (P < 0.05) (Fig. 8A). In order to evaluate the efficacy of rNDV expressing S protein of IBV in preventing shedding of virulent IBV in immunized chickens, at days 5 following challenge with virulent IBV, the tracheal swab samples collected from all chickens of each group were analyzed for the IBV specific lesions in chicken embryos. Our results showed that 2 out of 10 (20%) chickens vaccinated with rNDV expressing codon optimized S protein of IBV and 5 out of 10 (50%) chickens vaccinated with a commercial IBV vaccine were shedding virus in trachea, respectively, whereas 10 out of 10 (100%) of chickens immunized with parental rNDV and 5 out of 5 (100%) of unvaccinated chickens, infected with IBV, were shedding virus in the trachea (Fig. 8B).

### The protective efficacy of rNDVs against a highly virulent NDV challenge

**Figure 9:**
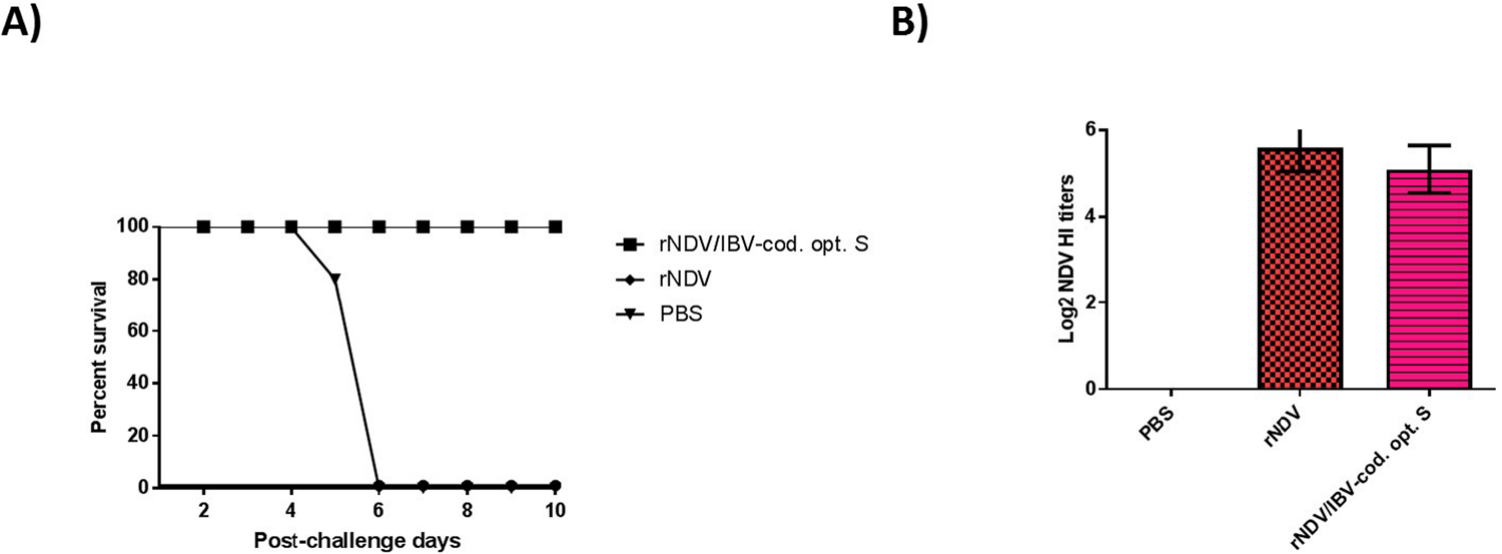
The protective efficacy of rNDVs against virulent NDV challenge and antibody responses against NDV in SPF chickens immunized at 1-day-old. **(A)** The protective efficacy of rNDV and rNDV expressing codon optimized S gene of IBV strain Mass-41 against highly virulent NDV strain Texas GB challenge. **(B)** HI antibody titers against NDV were assessed using HI assay. Serum titers are expressed as reciprocals of Log 2 dilution.

To evaluate the protective efficacy of rNDV expressing S gene of IBV against a virulent NDV strain, groups of five 1-day-old chicks were immunized with rNDV, rNDV expressing codon optimized S protein and PBS. Three weeks after immunization, chickens were challenged with virulent NDV strain Texas GB in our BSL-3 plus facility. Our results showed that all chickens immunized with the rNDV and rNDV expressing codon optimized S gene of IBV survived after highly virulent NDV challenge, while all chickens in PBS group died at day 5 and 6 post-challenge (Fig. 9A).

### Antibodies produced against IBV and NDV

Hemagglutination inhibition (HI) assay using a standard protocol of OIE was used to assess the level of antibodies mounted against NDV in serum samples of chickens 21 days after immunization. The results showed that HI titers of NDV was detected in serum samples of all chickens immunized with rNDV and rNDV expressing codon optimized S protein. There was no significant differences observed among HI titers against NDV in serum samples of chickens from groups immunized with rNDV and rNDV expressing S protein (Fig. 9B). Virus neutralization assay was performed according to a standard protocol of OIE to assess the level of neutralizing antibodies mounted against IBV strain Mass-41 in serum samples of chickens at 21 days after immunization. The results showed that the neutralizing antibodies against IBV were detected in serum samples of chickens immunized with rNDV expressing codon optimized S protein of IBV and with commercial live attenuated IBV vaccine (Fig. 7D). Neutralizing antibodies against IBV were not detected in 1:8 dilution of a serum sample from a chicken immunized with empty rNDV vector. This result showed that the rNDV expressing codon optimized S protein of IBV induces neutralizing antibodies against IBV.

## Discussion

This study was conducted to compare the protective efficacies of S1, S2, and S proteins of IBV using rNDV as a vaccine vector. The S1, S2, and S genes of IBV strain Mass-41 were individually inserted between the P and M genes of rNDV strain LaSota. This site was chosen because it has been identified as the optimal site for insertion of foreign genes into NDV genome^26,34–37^^.^ Four different versions of IBV S1 gene were used to identify the version that is expressed at the highest level and incorporated into NDV particles. We were able to recover all the recombinant viruses and their growth characteristics were similar to rLaSota. However, the recombinant viruses containing IBV S gene grew slightly slowly than the parental virus. The viruses were stable after passages in SPF chicken embryos. Western blot analysis showed that chicken codon optimized S2 and S proteins were expressed at much higher levels and were incorporated into NDV particles. Whereas, all the four versions of S1 protein were detected at very low levels by Western blot analysis. It is noteworthy that the unmodified S1 protein was detected in the infected cell culture supernatant, indicating that the modification of S1 protein probably caused retention of the protein in the cell. These results suggest that the S2 protein acts as a chaperone to assist in the folding of the S1 protein. The S1 protein is folded incorrectly in the absence of S2 protein and the new structure probably causes loss of some conformational epitopes for IBV antibodies.

In the first IBV protection experiment, we found that 1-day-old chicks immunized with rNDV expressing the S protein of IBV conferred better protection from disease compared to 1-day-old chicks immunized with rNDVs expressing either S1 or S2 protein of IBV. Our results showed that the S protein, which contains both S1 and S2 proteins, is the best protective antigen of IBV. The S2 protein lacks major neutralizing epitopes which are present in the S1 protein, hence it is not an effective antigen. The S1 protein contains major neutralizing epitopes, but it losses some conformational epitopes when expressed separately. Although in the first study we showed that rNDV expressing S protein provided enhanced protection, it could not reduce virus shedding, indicating that we needed to determine whether the elimination of virus shedding would probably require a much higher level of immune response than that is induced by rNDV expressing S protein or would require the optimization of the IBV protection study. In this study, our results support previous reports that rNDV vectored IBV vaccines prevent disease but do not stop virus shedding^23,24^. These results also support the recent report that a spike ectodomain subunit vaccine protects chickens against IBV^38^.

In the second IBV protection experiment, we investigated whether age at immunization influences the outcome of IBV challenge. Our results showed that a single immunization of 4-week-old chickens with rNDV expressing S protein completely protected chickens against IBV challenge based on disease and viral load in tracheas. Indeed, the level of protection conferred by rNDV expressing S protein was similar to that of a commercial IBV vaccine. However chickens immunized with either commercial live attenuated IBV vaccine or rNDV expressing IBV S protein showed very low levels of tracheal viral load. This showed that protection was greater when the chickens were immunized at an age when their immune system is relatively well developed.

In the third IBV protection experiment, we showed that rNDV expressing IBV S protein protects adult chickens against a higher dose of virulent IBV challenge. However, compared to standard challenge dose of virulent IBV, a higher challenge dose of virulent IBV caused higher levels of tracheal viral load in adult chickens immunized with rNDV expressing IBV S protein and low levels of tracheal viral load in chickens immunized with commercial live attenuated IBV vaccine. Our result showed that although both the age of immunization and dose of challenge virus affect the results of IBV challenge, the influence of the age of immunization is greater than the effect of the dose of challenge virus.

Our results also showed that when we challenged adult immunized chickens with standard challenge dose of virulent IBV, rNDV expressing S protein showed slightly better protection than a commercial live IBV vaccine, based on disease and viral shedding in trachea, but when the adult immunized chickens were challenged with a higher dose of virulent IBV, commercial live IBV vaccine showed slightly better protection than rNDV expressing S protein. Hence to compare the efficacy of rNDV expressing S protein of IBV with the efficacy of live attenuated IBV vaccine, a large IBV protection study using commercial chickens is needed.

In the fourth IBV protection experiment, we showed that rNDV expressing IBV S protein protected young chickens against virulent IBV challenge by the intraocular route. The route of challenge has been recommended by USDA-CFR-9. Our results showed that compared to infection of chickens with virulent IBV by the oculanasal route, infection of chickens with virulent IBV by the intraocular route, caused much lower levels of tracheal viral load in young chickens immunized with rNDV expressing IBV S protein and low levels of tracheal viral load in chickens immunized with commercial live attenuated IBV vaccine. Our results showed that the route of the challenge virus inoculation affected the results of the tracheal virus shedding in young chickens immunized by rNDV expressing S protein following IBV challenge; however, it did not affect the outcomes of the severity of clinical signs.

Although our studies showed that the rNDV expressing the S protein and commercial live IBV vaccine provided comparable protection, rNDV expressing the S protein has several advantages over live IBV vaccines in controlling IB in the field. (i) NDV vectored IBV vaccine is highly safe in 1-day-old chicks, (ii) it will not create new vaccine derived variant viruses, which is a major concern in using live modified IBV vaccines, (iii) a single vaccine can be used to control both NDV and IBV, (iv) we believe that the level of immunity induced by the NDV vectored vaccine against IBV is probably sufficient to completely stop IBV infection in field condition, and (v) the immune response of NDV vectored vaccine can be enhanced by prime-boost vaccination strategy.

In summary, we have shown that although the S1 and S2 proteins of IBV are known to contain virus neutralizing epitopes, the presence of the whole S protein is necessary for eliciting a strong protective immune response. The S protein is the antigen of choice for any vectored IBV vaccine. NDV is an attractive vaccine vector for IBV, because it can be used as a bivalent vaccine. Our results suggest that a recombinant NDV vectored IBV vaccine is the vaccine of choice for controlling IBV infection in the field.

## Material and Methods

### Cells and viruses

Chicken embryo fibroblast (DF-1) cells and human epidermoid carcinoma (HEp-2) cells were obtained from the American Type Culture Collection (ATCC, Manassas, VA). They were grown in Dulbecco’s minimal essential medium (DMEM) containing 10% fetal bovine serum (FBS). The recombinant avirulent NDV strain LaSota was generated previously in our laboratory using reverse genetics^39^. The rNDV and rNDVs expressing chicken codon optimized S1, S2 and S genes and non-codon optimized S gene of IBV strain Mass-41 were grown in 9-day-old embryonated SPF chicken eggs at 37 °C. The virulent IBV strain Mass-41 was propagated in 10-day-old SPF embryonated chicken eggs and harvested five days after infection. The titer of virus in harvested allantoic fluid was determined by 50% embryo infectious dose (EID_50_) method. Briefly, ten-fold serial dilutions of IBV strain Mass-41 was inoculated into 10-day-old embryonated SPF chicken eggs. Seven days after inoculation, infected embryos were examined for IBV specific lesions such as stunting or curling. The titer of virus was calculated using Reed and Muench method^40^.The modified vaccinia virus strain Ankara expressing T7 RNA polymerase (MVA-T7) was propagatd in monolayer primary chicken embryo fibroblast cells.

### Generation of rNDVs containing S1, S2 or S gene of IBV

A plasmid containing full-length antigenomic cDNA of NDV strain LaSota has been constructed previously^39^. In order to develop an effective IBV vaccine the maximum neutralizing epitopes with correct conformation are needed to be displayed. Most neutralizing epitopes are located in the S protein. In this study seven transcription cassettes containing S, S1 or S2 genes of IBV were constructed to identify the best protective antigen for the development of NDV vectored IBV vaccines. The S, S1 and S2 genes were chicken codon optimized for higher level of expression in chickens. The following transcription cassettes were designed: (i) a transcription cassette containing the S gene of IBV strain Mass-41 (3489 nt) was designed to determine whether the expression of the whole S gene from NDV will lead to display the maximum neutralizing epitopes in correct conformation, (ii) a transcription cassette containing the S2 subunit of S gene (1878 nt) of IBV fused with C-terminus of signal peptide sequence of S gene (69 nt) was constructed for transport of the protein from the cell (iii) a transcription cassette containing the S1 subunit of S gene (1614 nt) was designed to determine the protective efficacy of S1 protein, (iv) a transcription cassette containing the S1 gene (1611 nt) fused with N-terminus of transmembrane and cytoplasmic tail of S gene (255 nt) was designed for incorporation into NDV envelop, (v) a transcription cassette containing the S1 subunit of S gene without S1 protein cleavage site residues (1593 nt) fused with N-terminus of transmembrane and cytoplasmic tail of NDV F gene (171nt) was designed for incorporation of the S1 protein into envelope of NDV. (vi) a transcription cassette containing the S1 subunit of S gene containing S1 protein cleavage site residues (1611 nt) fused with N-terminus of transmembrane and cytoplasmic tail of NDV F gene (171 nt) was designed to incorporate the S1 protein into NDV envelope and also to know whether adding the cleavage site residues has any effect on the fusion of two proteins, and (vii) a transcription cassette containing the non-codon optimized S gene (3489 nt) was constructed to compare the level of protein expression between the codon optimized and non-codon optimized S genes.

NDV genome contains six genes: nucleocapsid(N), phosphoprotein(P), matrix(M), fusion(F), hemagglutinin-neuraminidase(HN) and large(L). The genes are ordered 3′-N-P-M-F-HN-L-5′. The beginning and the end of each gene contain conserved transcriptional sequences known as the gene-start (GS) and gene-end (GE), respectively. Between the genes, there are gene junctions^26^. Any of the gene junctions is a potential insertion site for the transcription cassette of a foreign gene. However, we and others have found that the intergenic region between the P and M genes is a good site for expression of most foreign genes^26,34–37^. The transcription cassettes containing IBV genes contained *PmeI* restriction enzyme sequence, 15 nt of untranslated region (UTR) of NDV, NDV GE signal, one T nucleotide as intergenic sequence, NDV GS signal, extra nucleotides to maintain the rule of six^26,41^, Kozak sequence at the upstream of foreign gene ORFs and *PmeI* restriction enzyme sequence at downstream of foreign gene ORF. The transcription cassettes of codon optimized and non-codon optimized S gene were digested from two commercially synthesized (GenScript; pUC57-IBV-Mass-41-S syn) plasmids containing codon optimized (GenScript; optimization on *Gallus Gallus* codons using OptimumGene^TM^ PSO algorithm) and non-codon optimized S gene of IBV strain Mass-41(GenBank Accession no. AY851295. 1), respectively. The transcription cassettes of codon optimized S1 and S2 genes were amplified from the commercially synthesized plasmid containing codon optimized S gene of IBV strain Mass-41 and cloned into individual shuttle vectors (pGEM®-T Easy Vector, Promega Corporation). Then the flanking DNA of transcription cassettes were digested from shuttle vectors. The transcription cassettes derived from shuttle vectors were cloned into complete individual plasmids containing cDNA of rLaSota at P and M gene junction using *PmeI* site (Fig. 1). The correct sequences of the foreign genes were confirmed by nucleotide sequence analysis. rNDVs containing the IBV genes were recovered by reverse genetics as described previously^39^.Briefly, each full length cDNA was co-transfected with three expression plasmids containing N, P or L gene of NDV strain LaSota into MVA-T7 infected HEp-2 cells. Three days post-transfection, 200 µl of supernatant of transfected cells were inoculated in 9–11 day-old SPF embryonated chicken eggs. After three days, haemaglutination test was used to detect infected allantoic fluids collected from eggs. rNDVs containing S1 gene, S1 gene fused with transmembrane and cytoplasmic tail of IBV S gene, S1 gene containing cleavage site residues of S gene of IBV fused with transmembrane and cytoplasmic tail of NDV F gene, S1 gene without cleavage site residues of S gene fused with transmembrane and cytoplasmic tail of NDV F gene, S2 gene, codon optimized S gene and non-codon optimized S gene were named rNDV/S1, rNDV/S1 + IBV-S-TM&CT, rNDV/S1(cs+) + NDV-F-TM&CT, rNDV/S1(cs−) + NDV-F-TM&CT, rNDV/S2, rNDV/codon optimized-S and rNDV/non-codon optimized-S, respectively. The IBV genes were amplified from the rNDV constructs by RT-PCR.

### Expression of S1, S2 and S proteins of IBV

Confluent monolayers of DF-1 cells were infected at a multiplicity of infection (MOI) of 0.01–0.1 with rNDV strain LaSota, rNDV/S1, rNDV/S1 + IBV-S-TM&CT, rNDV/S1(cs+) + NDV-F-TM&CT, rNDV/S1(cs−) + NDV-F-TM&CT, rNDV/S2, rNDV/codon optimized-S or rNDV/non-codon optimized-S. DF-1 cells were harvested 30 hours post-infection, lysed and analyzed by Western blot. A polyclonal chicken anti-IBV strain Mass-41 was used to detect the expression of S1, S2 and S proteins of IBV. To determine the incorporation of IBV proteins into NDV envelope, rNDV, rNDV/S2, rNDV/codon optimized-S and rNDV/non-codon optimized-S were inoculated into 9-day-old embryonated SPF chicken eggs. Three days after incubation, recombinant viral particles from infected allantoic fluids were partially purified by sucrose density gradient centrifugation and analyzed by Western blot analysis. A monoclonal anti-NDV/HN antibody also was used to detect HN protein of NDV in lysates and purified virions by one more Western blot analysis.

### Growth characteristics of rNDV constructs

In order to determine the growth kinetics of rNDVs expressing S1, S2 or S protein of IBV, confluent monolayers of DF-1 cells in 6-well tissue culture plates were infected at a MOI of 0.1 with rNDV, rNDV/S1(cs+) + NDV-F-TM&CT, rNDV/S2 and rNDV/codon optimized-S and adsorbed for 90 minutes at 37 °C. After adsorption, cells were washed with PBS, then incubated with DMEM containing 2% FBS and 10% fresh SPF chicken egg allantoic fluid at 37 °C in presence of 5% CO_2_. Aliquots of 200 µL of supernatant from infected cells were collected and replaced with fresh DMEM including FBS at intervals of 8 hours until 64 hours post-infection. The titer of virus in the harvested samples was determined by TCID_50_ method in DF-1 cells in 96-well tissue culture plates.

### The protective efficacy of rNDVs expressing S1, S2 and S protein of IBV against virulent IBV challenge

Based on the level of expression of S1, S2 and S proteins of IBV from rNDVs, rNDV/S1(cs+) + NDV-F-TM&CT, rNDV/S2, and rNDV/codon optimized-S viruses were selected for animal study to evaluate their protective efficacy against virulent IBV challenge.

### IBV protection experiment 1

In this study, the protective efficacy of rNDVs expressing S1, S2 or S protein of IBV strain Mass-41 were evaluated in 1-day-old SPF chicks. Briefly, a total of eighty 1-day-old chicks were divided into five groups of fifteen each and one group of five. Chicks of the first four groups were inoculated with 10^7^ EID_50_ of rNDV, rNDV/S1(cs+) + NDV-F-TM&CT, rNDV/S2 and rNDV/codon optimized-S strains via oculonasal route. The fifteen chicks of group five and five chicks of group six were inoculated with PBS. Three weeks after immunization, all immunized chickens, were challenged with 10^3.1^ EID_50_ of virulent IBV strain Mass-41. This challenge virus dose was determined by an experimental chicken infection study. The severity scores of clinical signs of IBV including, nasal discharge, ocular discharge and difficulty in breathing (0 = normal, 1 = presence of mild ocular discharge, mild nasal discharge and or sneezing 2 = presence of heavy ocular discharge and or heavy nasal discharge with mild tracheal rales and mouth breathing and or coughing 3 = heavy ocular discharge and heavy nasal discharge with sever tracheal rales and mouth breathing, gasping, dyspnea and or severe respiratory distress) were recorded twice a day for 10 days post-challenge. In order to evaluate protective efficacy of rNDVs expressing S1, S2 and S genes of IBV in preventing shedding of virulent IBV in immunized chickens, at day five post-challenge, tracheal swab samples were collected from fifteen birds of each group and placed in 1.5 mL serum free DMEM with 10 X antibiotics. The swab samples were analyzed for quantification of viral RNA using an IBV-N gene-specific RT-qPCR.

### IBV protection experiment 2

In this study, the protective efficacy of rNDV expressing codon optimized S protein of IBV was evaluated in 4-week-old SPF chickens against the OIE recommended dose of virulent IBV challenge^1^. A total of twenty 4-week-old SPF chickens were divided into four groups of five each. Five chickens of groups one and two were inoculated with 10^7^ EID_50_ of rNDV and rNDV/codon optimized-S, respectively, via oculanasal route. Five chickens of group three were inoculated with 10 recommended doses of a commercial live attenuated Mass-type IBV vaccine via oculanasal route and chickens of group four were inoculated with PBS. Three weeks after immunization, chickens of all groups were challenged with 10^3.1^ EID_50_ of virulent IBV strain Mass-41 by the oculonasal route. The severity scores of clinical signs of IBV, described in IBV protection experiment 1, were recorded for 10 days post-challenge. In order to evaluate the efficacy of rNDV expressing S protein of IBV in preventing shedding of virulent IBV in immunized chickens, at day 5 post-challenge, tracheal swab samples were collected from twenty chickens and placed in 1.5 ml serum free DMEM with 10 X antibiotic. The swab samples were analyzed for quantification of viral RNA using an IBV-N gene-specific RT-qPCR.

### IBV protection experiment 3

In this study, the protective efficacy of rNDV expressing codon optimized S protein of IBV was evaluated in 4-week-old SPF chickens against a higher dose of virulent IBV challenge. A total of thirty two 4-week-old SPF chickens were divided into four groups of eight each. Eight chickens of group one and two were inoculated with 10^7^ EID_50_ of rNDV and rNDV/codon optimized-S, respectively, via oculanasal route. Eight chickens of group three were inoculated with 10 recommended doses of a commercial live attenuated Mass-type IBV vaccine via oculanasal route and chickens of group four were inoculated with PBS. Three weeks after immunization, chickens of all groups were challenged with 10^4.7^ EID_50_ of virulent IBV strain Mass-41 by the oculonasal route. The severity scores of clinical signs of IBV, described in IBV protection experiment 1, were recorded for 8 days post-challenge. At day 4 post-challenge, three chickens from each group were euthanized for tracheal ciliostasis analysis (data not shown). In order to evaluate the efficacy of rNDV expressing S protein of IBV in preventing shedding of virulent IBV in immunized chickens, tracheal swab samples were collected from five chickens from each group and placed in 1.5 mL serum free DMEM with 10 X antibiotic. Each fluid was tested for IBV specific lesions on chicken embryo by inoculation (0.1 ml) of one 10-day-old embryonated SPF chicken egg. The swab samples were also analyzed for quantification of viral RNA using an IBV-N gene-specific RT-qPCR. The swab samples collected from two non-vaccinated SPF chickens involved in another IBV protection study also were used as control.

### IBV protection experiment 4

In this study, the protective efficacy of rNDV expressing codon optimized S protein of IBV was evaluated in 1-day-old SPF chicks against virulent IB challenge virus infected by the intraocular route. Intraocular route was used, because this route of IBV challenge has been specified by the USDA-CFR-9^33^. A total of forty five 1-day-old SPF chickens were divided into three groups of ten each and three groups of five each. Ten chickens of group one and two were inoculated with 10^7^ EID_50_ of rNDV, rNDV/codon optimized-S, respectively, via oculanasal route. Ten chickens of group three were inoculated with one recommended dose of a commercial live attenuated Mass-type IBV vaccine via oculanasal route and chickens of groups four to six were left non-vaccinated. Three weeks after immunization, chickens of all groups one to four were challenged with 10^4^ EID_50_ of virulent IBV strain Mass-41 by the intraocular route, chickens of group five were challenged with 10^4^ EID_50_ of virulent IBV strain Mass-41 by the oculanasal route, and chickens of group six were left non-infected. The severity scores of clinical signs of IBV, described in IBV protection experiment 1, were recorded for 10 days post-challenge. In order to evaluate the efficacy of rNDV expressing S protein of IBV in preventing shedding of virulent IBV in immunized chickens, at day 5 post-challenge, tracheal swab samples were collected from all chickens of each group and placed in 3 mL serum free DMEM with 10 X antibiotic. Each fluid was tested for IBV specific lesions on chicken embryo by inoculation with 0.2 ml to each of five 10-day-old embryonated SPF chicken egg. The sample was considered positive for virus shedding, if any of the five embryos showed IBV lesions.

We performed all experiments involving virulent IBV in our USDA approved Biosafety level-2 and Biosafety level-2 plus facilities following the guidelines and approval of the Animal Care and Use Committee (IACUC), University of Maryland.

### The protective efficacy of rNDV expressing S protein of IBV against virulent NDV challenge

The protective efficacy of rNDV expressing S protein of IBV strain Mass-41 was evaluated against a virulent NDV strain GB Texas challenge in our biosafety level 3 (BSL-3) plus facility. Briefly, a total of fifteen 1-day-old chicks were divided into three groups of five each. Chicks of two groups were inoculated with 10^7^ EID_50_ of rNDV and rNDV/IBV-codon optimized-S via oculonasal route. The five chickens of group three were inoculated with PBS. Three weeks after immunization, blood samples of all birds were collected for NDV antibody response analysis and challenged with one hundred 50% chicken lethal dose (CLD_50_) of the highly virulent NDV strain GB Texas via oculonasal route. The chickens were observed daily for 10 days after challenge for mortality with clinical signs of disease (neurological signs included torticollis, paralysis, and prostration). We performed the experiment involving virulent NDV in our USDA approved Biosafety level-3 plus facility following the guidelines and approval of the Animal Care and Use Committee (IACUC), University of Maryland.

### Serological analysis

The level of antibodies induced against NDV and IBV were evaluated. The serum samples were collected three weeks post-immunization. Hemagglutination inhibition (HI) assay using a standard protocol OIE was used to assess the level of antibody titer mounted against NDV in chickens immunized by rNDVs^27^. The virus neutralization assay according to OIE was used to measure the level of neutralizing antibodies mounted against IBV^1^. Briefly, serum samples of three birds from the group immunized with rNDV expressing codon optimized S protein of IBV and serum samples of three birds from the group immunized with commercial IBV vaccine group were incubated at 56 °C for 30 minutes. One hundred EID_50_ of IBV strain Mass-41 was mixed with 2 fold dilutions of antiserum and incubated for 1 hour at 37 °C. One hundred µL of each serum and virus mixture was inoculated into three 10-day-old embryonated SPF chicken eggs. To confirm that at least 100 EID_50_ of virus was inoculated into each egg, three eggs were inoculated with 100 µl of PBS containing 100 EID_50_ of IBV. Three eggs were inoculated with 100 µL of PBS as negative control. Three eggs were inoculated with a mixture of 100 EID_50_ of IBV and a dilution of 1:8 of a randomly selected serum sample collected from a bird immunized with rNDV strain LaSota as vector control. The eggs were incubated at 37 °C and were observed daily for dead chicken embryos for 7 days post inoculation. The serum titers were calculated according to the method of Reed and Muench^40^, based on mortality and IBV specific lesions on chicken embryos.

### Quantitative reverse transcription-polymerase chain reaction (RT-qPCR)

RNA was extracted using TRIzol Reagent (Invitrogen) from tracheal swab samples collected from chickens. The first strand cDNA was synthesized using Thermo Scientific RevertAid Reverse Transcriptase (RT). SYBR green RT-qPCR was performed using a specific primer pair set: (a) N gene - 296 forward primer: 5′ GACCAGCCGCTAACCTGAAT 3′ and (b) N gene - 445 reverse primer: 5′ GTCCTCCGTCTGAAAACCGT 3′ amplifying 150 nt of N gene of IBV strain Mass-41. PCRs were performed using a Bio-Rad CFX96 Cycler. Each 20 µl reaction was carried out using 5 µl of cDNA, 10 µl of iTaq Universal SYBR Green Supermix (Bio-Rad), 2 µl of forward and reverse primers and 3 µl of nuclease free water. Forty cycles of PCR at 95 °C for 10 s (denaturation), 58 °C for 20 s (annealing), and 72 °C for 30 s (elongation) followed by melting curve analysis that consisted of 95 °C for 5 s and 65 °C for 60 s. A serial 10 fold dilution of cDNA synthesized from extracted RNA of allantoic fluid stock of a virulent IBV strain Mass-41 with 10^7.5^ EID_50_/ml was used to establish the standard curve. The cDNA synthesized from extracted RNA of allantoic fluid stock of a virulent IBV strain Mass-41 and the cDNA synthesized from extracted RNA of swab sample solution were served as positive and negative controls, respectively. Melting point analysis was used to confirm the specificity of the test.

### Statistical analysis

Data were analyzed among groups by One-Way-ANOVA test. The student *t*-test was used to compare two groups. To avoid bias, all animal experiments were designed as blinded studies.

## Electronic supplementary material

Supplementary data
