# Author Correction: A Recombinant Newcastle Disease Virus (NDV) Expressing S Protein of Infectious Bronchitis Virus (IBV) Protects Chickens against IBV and NDV

**DOI:** 10.1038/s41598-020-57826-w

**Published:** 2020-01-15

**Authors:** Edris Shirvani, Anandan Paldurai, Vinoth K. Manoharan, Berin P. Varghese, Siba K. Samal

**Affiliations:** Virginia-Maryland College of Veterinary Medicine, University of Maryland, College Park, MD USA

Correction to: *Scientific Reports* 10.1038/s41598-018-30356-2, published online 10 August 2018

This Article contains errors in the Methods under subheading ‘IBV protection experiment 2’.

“A total of twenty 4-week-old SPF chickens were divided into four groups of five each. Five chickens of groups one and two were inoculated with 10^7^ EID_50_ of rNDV and rNDV/codon optimized-S, respectively, via oculanasal route. Five chickens of group three were inoculated with 10 recommended doses of a commercial live attenuated Mass-type IBV vaccine via oculanasal route and chickens of group four were inoculated with PBS.”

should read:

“A total of fifteen 4-week-old SPF chickens were divided into three groups of five each. Five chickens of groups one and two were inoculated with 10^7^ EID_50_ of rNDV/codon optimized-S and 10 recommended doses of a commercial live attenuated Mass-type IBV vaccine via oculanasal route, respectively. Chickens of group three were inoculated with PBS. Samples collected from five non-vaccinated SPF chickens involved in another IBV protection study also were used as control.”

Furthermore, within the Methods under subheading ‘Quantitative reverse transcription-polymerase chain reaction (RT-qPCR)’

“Forty cycles of PCR at 95 °C for 10 s (denaturation), 58 °C for 20 s (annealing), and 72 °C for 30 s (elongation) followed by melting curve analysis that consisted of 95 °C for 5 s and 65 °C for 60 s.”

should read:

“The thermal conditions of 50 °C for 10 min and 95 °C for 10 min, then forty-five cycles of PCR at 95 °C for 10 s (denaturation), 58 °C for 20 s (annealing) followed by 95 °C for 5 s and melting curve analysis that consisted of 65 °C to 95 °C for 5 s was carried out to amplify the 150 nt – N gene fragments.”

