# Supplementary material for: A Recombinant Newcastle Disease Virus (NDV) Expressing S Protein of Infectious Bronchitis Virus (IBV) Protects Chickens against IBV and NDV
Source: Sci Rep. 2018 Aug 10;8:11951. doi: 10.1038/s41598-018-30356-2 (PMC6086832; doi:10.1038/s41598-018-30356-2)
Supplement: Supplementary file 1 — Supplementary data [file 41598_2018_30356_MOESM1_ESM.docx]

**Supplementary Information**

A Recombinant Newcastle Disease Virus (NDV) Expressing S Protein of Infectious Bronchitis Virus (IBV) Protects Chickens against IBV and NDV.

Edris Shirvani, Anandan Paldurai, Vinoth K. Manoharan, Berin P. Varghese and Siba K. Samal

Virginia-Maryland College of Veterinary Medicine, University of Maryland, College Park, MD, USA.


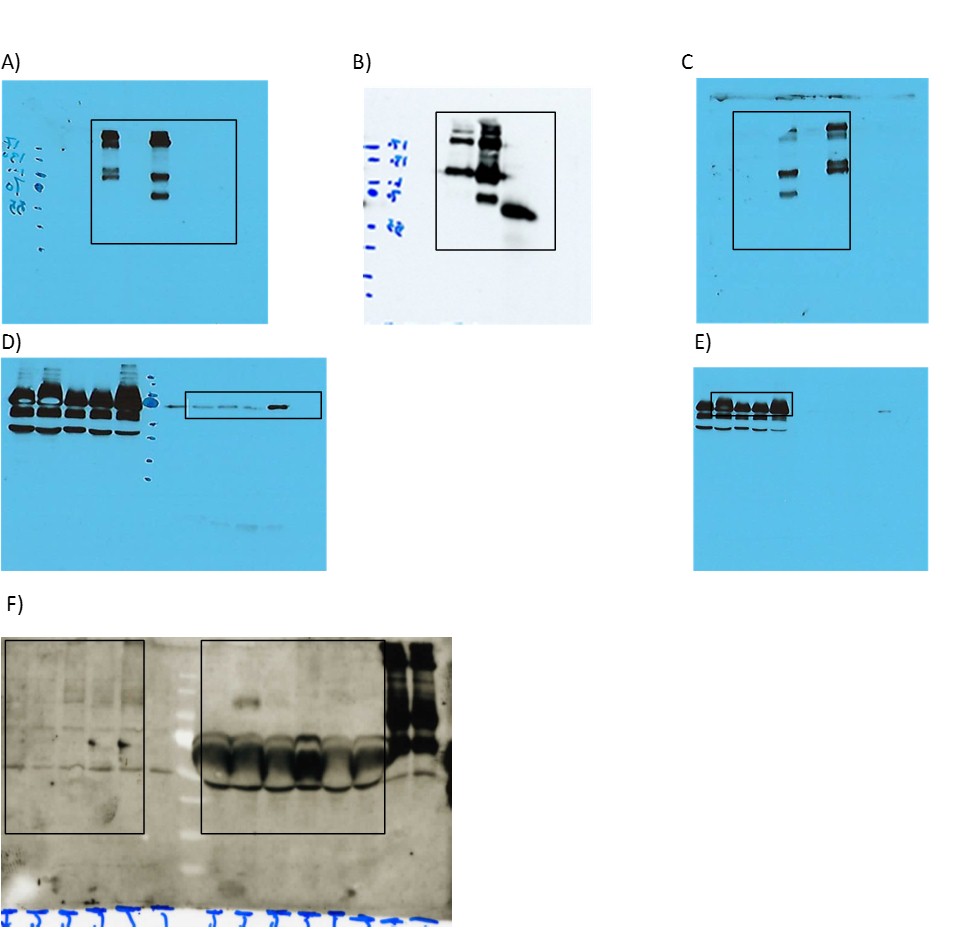


**Supplementary figure S1:**  Full length gels of cropped versions of gels which were shown in the main text (A-F). A, B, C, D, E and F represent gels in fig. 2A- upper panel, fig. 2B, fig. 2C-upper panel, fig. 2A lower panel, fig. 2C lower panel and fig.3, respectively. The cropped bands are shown with black boxes.
